# Changes in Garlic Quality during Fermentation under Different Conditions

**DOI:** 10.3390/foods13111665

**Published:** 2024-05-26

**Authors:** Aurelija Paulauskienė, Šarūnas Kulbokas, Egidijus Zvicevičius, Živilė Tarasevičienė

**Affiliations:** 1 Department of Plant Biology and Food Sciences, Vytautas Magnus University Agriculture Academy, Faculty of Agronomy, Studentų st. 15, Kaunas District, LT-53361 Akademija, Lithuania; zivile.taraseviciene@vdu.lt; 2 Department of Biology, Vytautas Magnus University Faculty of Natural Sciences, Universiteto str. 10, Kaunas District, LT-53361 Akademija, Lithuania; saru.kulb@gmail.com; 3 Department of Mechanical, Energy and Biotechnology Engineering, Vytautas Magnus University Agriculture Academy, Faculty of Engineering, Studentų st. 15, Kaunas District, LT-53361 Akademija, Lithuania; egidijus.zvicevicius@vdu.lt

**Keywords:** garlic, antioxidant activity, fermentation, phenolic compounds, physical properties

## Abstract

One of the garlic processing methods is fermentation, which produces black garlic with completely different chemical, physical, sensory, culinary, and health-enhancing properties. Our study aimed to compare the influence of various processing conditions on the quality indicators of black garlic (BG). Samples of white garlic (WG) were placed in laboratory climatic chambers. BG1 samples were packed in plastic bags and vacuumed, BG2 and BG3 samples were packed in textile mesh bags. BG1 samples were fermented in 70% humidity at 50 °C for 28 days, BG2 samples in 85% humidity at 60 °C for 99 days, and BG3 samples in 80% humidity at 80 °C for 14 days. The dependence of changes in chemical composition, color, and texture of garlic on fermentation conditions was analyzed. Proximate composition analyses and antioxidant activity of WG and BG were performed using standard methods. It was established that regardless of the fermentation conditions, BG’s chemical composition became richer than WG’s. They significantly increased vitamin C content (1.5–5.8 fold), titratable acidity (14.7–21.0 fold), protein (1.4–3.2 fold), fiber (4.6–7.0 fold), and ash (1.2–3.9 fold) content, amount of total phenolic compounds (6.6–9.6 fold) and antioxidant activity (5.3–9.9 fold). Fermented garlic turned dark in color and soft and sticky in texture. The higher fermentation temperature (80 °C) but the shorter time (14 days) had the greatest positive effect on the quality of black garlic.

## 1. Introduction

For centuries, garlic (*Allium sativum* L.) has been widely cultivated and used as a spice and traditional medicinal plant against various diseases. Numerous studies have demonstrated that garlic has a wide range of therapeutic effects, such as anticancer, hypolipidemic, antihypertensive, hepatoprotective, and immunomodulatory effects, attributed to its bioactive compounds [1,2,3,4]. The health properties of garlic are determined by the bioactive compounds, in particular organosulphur compounds, especially thiosulfinates [4,5]. Although noted for its various medicinal properties, garlic should be used in moderation, as excessive consumption can damage the lining of the stomach and intestines, cause anemia and contact dermatitis, and decrease serum protein and calcium levels [1,6]. In addition, the strong, pungent smell of raw garlic penetrates even through human skin and causes an unpleasant body and mouth odor [4,6,7]. To reduce or eliminate the unpleasant smell while maintaining or increasing the beneficial properties of garlic, various processing technologies have been used in recent years, such as heat treatment or fermentation, also known as aging [1,6,7].

Aged garlic, which is also called black garlic (BG), is produced by fermentation of raw white garlic (WG) at high temperatures (40–90 °C) under high humidity (60–90%) for a period of time (10–90 days). Heat treatment triggers many chemical reactions in garlic. Enzymatic browning and the Maillard reaction intermediate the Amadori and Heyns change of garlic color from white and yellow to dark brown [8,9]. During the heat treatment of BG, complicated compounds are formed, and the quality of the final product depends on the selected manufacturing conditions, but the BG contains more functional compounds than fresh garlic [9]. During heating, unstable, unpleasant, and pungent compounds in raw garlic are converted into stable, tasteless compounds. This is caused by the change of the allicin, which contributes to the characteristic odor and taste of garlic, into water-soluble antioxidants, including S-allylcysteine (SAC), tetrahydro-β-carbolines, biologically active alkaloids, and flavonoids [8,9,10]. Importantly, fermented garlic is found to have increased levels of SAC, which is believed to be responsible for the attractive health-promoting properties of BG, such as antidiabetic, antioxidant, and anti-inflammatory activities [2,4,9]. Researchers stated that the SAC in black garlic can reduce oxidative damage and the possibility of various diseases such as cardiovascular changes, cancer, stroke, Alzheimer’s disease, and other degenerative diseases related to age [7,11].

Garlic has a high content of free amino acids, playing a significant role in the Maillard reaction during the thermal treatment of WG. Lactic acid bacteria that occur in fermented products can decompose the protein to produce a variety of small peptides, especially the essential amino acids and free amino acids. Therefore, improves the digestibility of protein in food and enhances the nutritional value of protein. Researchers detected a remarkably higher cysteine content in BG samples than in WG [12]. On the other hand, the cysteine content in BG decreased during heat treatment [8]. In the aging period, the decrease of cysteine and tyrosine might be related to Maillard reactions, which usually occur between amino acids and reducing sugars. This can be considered one of the key findings regarding the changes in the amino acid composition during the fermentation process.

According to several studies, carbohydrate content can increase by one- to twofold in BG compared to WG [11]. During heat treatment and related to processing temperature, fructans break into monosaccharides, disaccharides, and oligosaccharides [13,14,15]. Fructans level during garlic processing may decrease by around sixfold, and monosaccharides in BG become the predominant saccharides [16,17]. BG reducing sugar content increases 20–70-fold compared with WG, which causes a sweet taste of the product [18,19]. In processing food by fermentation, the degradation of polysaccharides by lactic acid bacteria can produce monosaccharides, lactic acid, and other compounds, which improves the food quality [15,17]. 

Research data shows that the fermentation process changes the chemical composition and improves the bioactivity of BG. Lactic acid bacteria play a major role in these changes. Lactic acid bacteria can break down macromolecular food substances, including indigestible polysaccharides, and transform undesirable or unpleasant odorous substances [20]. The aroma and taste of BG changes largely due to their metabolism. 

In fermented foods, lactic acid bacteria produce antioxidant substances that can have a variety of beneficial effects on human health., BG contains many more functional compounds than fresh ones [8,12,21,22].

The heating process of garlic leads to the formation of biological compounds such as phenolics [21,23]. The amount of phenolic compounds in BG is closely related to the selected fermentation method. The total phenolic content of BG varies between 258 and 812 mg GAE 100 g^−1^ DM [8,12,21,24,25,26]. According to Kim et al. [21], phenolic acid contents in BG increased over fivefold compared to WG and is a significant source of phenolic acids (coumaric, ferulic, and cafeic), flavonols (myricetin, resveratrol, morin, and quercetin), and flavanols (epicatechin, catechin, and epicatechin gallate). 

During processing at different temperatures and humidity, garlic increases the content of total water-soluble vitamins [18]. Kim et al. [27] stated that the total water-soluble vitamin content increased by about 1.15–1.92 fold in BG to compare with WG.

Scientists have determined that BG has a higher radical scavenging capacity than raw garlic, and fermentation enhances the antioxidant properties [22]. Antioxidant capacity increases during heat treatment of garlic due to the formation of new antioxidant constituents such as hydroxymethylfurfural, SAC, polyphenols, melanoidins, carboline derivatives, Heyns and Amadori compounds [12].

Consuming foods high in polyphenols and antioxidant capacity over the long term may offer protection from developing cardiovascular and degenerative diseases, cancer, and diabetes, according to evidence from epidemiological studies [28,29,30,31,32,33,34].

Studies show that the contents of chemical compounds of BG depend on the conditions during thermal processing. Research data confirms that many valuable compounds increase during garlic heat treatment, especially polyphenols, flavonoids, and some intermediate components of the Maillard reaction known as antioxidant agents [9].

Many studies analyze the chemical composition, biologically active substances, functional properties of BG, and their effects on human health. However, we have found only some results of studies comparing the effectiveness of fermentation conditions on the chemical composition of BG. Choi et al. [8] suggested that 70 °C, 90% relative humidity and 21 days of fermentation are the optimal conditions for BG production. Zhang et al. [18] stated that good quality and flavor of BG occur under 70 °C fermentation conditions. Our study aimed to determine the influence of packaging, temperature, relative humidity and fermentation process duration on BG’s chemical composition and physical properties.

## 2. Materials and Methods

### 2.1. Plant Materials

The object of research was fresh white garlic (*Allium sativum* L.) (WG) and fermented black garlic (BG1, BG2, BG3). Garlic was grown on an organic farm in the Radviliskis district., Lithuania (55.63934° N, 23.72362° E).

WG samples in five replicates were placed in laboratory climatic chambers Feutron KPK 600 (Feutron GmbH, Langenwetzendorf, Germany) with different humidity and temperature and fermented at distinctive times (Table 1). Since thermal treatment temperature and relative humidity during BG production are critical parameters that most influence BG quality [34], completely different garlic fermentation conditions were chosen for the experiment. BG1 garlic samples (6.0 ± 0.035 kg) were packed in 450 mm × 500 mm plastic bags of 9-layer PA/PE film, vacuumed with LAVA V400 Premium Vacuum Packing Machine (Landig Group, Bad Saulgau, Germany) and placed in the climatic chamber. BG2 (5.9 ± 0.022 kg) and BG3 (5.9 ± 0.022 kg) garlic samples were packed in 520 mm × 800 mm textile mesh bags and put in the climatic chambers. 

The bags in the climate chambers were actively ventilated by air circulating in a circle. Fermentation temperature and humidity were automatically monitored by a sensor installed in the chamber, and an additional mobile resistance thermometer was placed between the bags with fermented garlic samples. During the fermentation, the condition of the garlic bulbs was periodically checked: visually evaluated the color of the skins of the bulbs and the cross-section of randomly taken bulbs’ color and structure of the garlic pulp was performed. Such visual evaluations were not performed with BG1 garlic. The vacuum package was cut open, and the pulp status of BG1 garlic was first evaluated 14 days after the start of fermentation.

After the garlic pulp acquired the desired color and texture, the fermentation process was stopped. In a suitable climatic chamber for 24 h, the temperature was reduced to 35 °C and the relative humidity was reduced to 30%. After the garlic has cooled down and the skin moisture has decreased, a sample of fermented garlic (about 1 kg) is randomly collected from the package, placed into a polyethylene box and stored in a refrigerator at 5 °C until analyzed. 1.0 ± 0.25 kg laboratory samples were composed of all fermentation replicates.

### 2.2. Determination of Chemical Composition

WG and BG samples were analyzed. Analytical grade chemicals used in this study were purchased from Labochema LT (Vilnius, Lithuania). Chemical analyses were performed in triplicate.

#### 2.2.1. Proximate Composition Analyses

Dry matter (DM) content was assessed by drying the shredded WG and BG samples to a constant mass at 105 °C [35]. 

About 1 kg of each laboratory sample of WG, BG1, BG2, and BG3 bulbs were divided into cloves that were peeled and homogenized with adaptable homogenizer VDI 25 (VWR International GmbH, Darmstadt, Germany). 

Ascorbic acid content (AAC) was determined by titration with 2.6-dichlorphenol-indophenol sodium salt dehydrate [35]. 

Titratable acidity (TA) was determined by titration with 0.1 N sodium hydroxide solution in the presence of phenolphthalein as an indicator of the pink color and expressed as % of lactic acid [35]. 

The amount of crude protein (CP) was estimated by the Kjeldahl method [36]. 

The amount of dietary fiber (DF) was determined using a modified version of the Henneberg–Stohmann method [37]. 

The amount of crude ash (CA) was determined by the dry burning of samples at a temperature of 500 °C in muffle furnace L9/11 (Nabertherm GmbH, Lilienthal, Germany) [38].

The total phenolic content (TPC) was established using the Folin–Ciocalteu reagent [39]. 10 mL of 75% ethanol (*v*/*v*) was added to 0.1 g of homogenized BG sample and extracted in the ultrasonic bath AU65 (Cromservis s.r.o., CZ) in 20 °C water for 30 min. Then, the extract was centrifuged in Clinispin Horizon 755VES centrifuge (Woodley Trial Solutions, Bolton, UK) at 3000 rpm for 10 min. At 20 °C, 0.2 mL of prepared extract was mixed with 0.2 mL of the Folin–Ciocalteu reagent (concentration 1.9–2.1 N), after which 1 mL of sodium carbonate (20%) was added and the mixture was completed to 5 mL with pure water. After 30 min of incubation at 20 °C in the dark, the absorbance was measured at 765 nm using a UVD-3200 spectrophotometer (Labomed Inc., Los Angeles, CA, USA). Total phenolic content was measured with the calibration curve by using gallic acid equivalent standards. The results were expressed as equivalents of gallic acid (mg GAE 100 g^−1^).

Total phenolic content calculated according to the following equation [39]:

(1) $$GRE=C\;\frac{V}{m}\times 100\;,$$

where *C*—gallic acid concentration (mg mL^−1^) is determined from the calibration curve, *V*—volume of the extract (mL), and *m*—mass of sample (g). 

The WG sample was prepared under the same conditions, except that 1 g of sample was used.

#### 2.2.2. The Antioxidant Activity Analysis

The antioxidant activity (AA) was determined using the 2.2-diphenyl-1-picrylhydrazyl (DPPH) free radical scavenging method [40]. One gram of homogenized WG and BG was mixed with 10 mL of 95% methanol and shaken for 30 min in a Vibramax 100 shaker (Heidolph Instruments GmbH & Co. KG, Schwabach, Germany), then centrifuged for 10 min at 3500 rpm at 20 °C. An amount of 3 mL 0.1 mM of DPPH methanol solution was added to the 0.1 mL of an aliquot of the methanolic extract. It was incubated in the dark at room temperature for 30 min. The absorbance was measured at the 517 nm wavelength. Antioxidant activity was calculated according to the formula and expressed in percentages:

(2) $$DPPH=\frac{A_{0}-A_{1}}{A_{0}}\times 100,$$

where *A*_1_—absorbance of the sample, *A*_0_—absorbance of the blank sample.

### 2.3. Determination of Physical Properties

#### 2.3.1. Color Analysis 

WG and BG peeled cloves were used for color and texture analysis.

The color coordinates of lightness, redness and yellowness L*, a*, and b*, according to the CIELAB scale, were determined by the ColorFlex color analyzer (HunterLab, Reston, VA, USA). A glass sample cup filled with peeled cloves was placed at the measurement port. The opaque cover was placed over the filled cup at the sample port.

The L* value indicates brightness ranging from black (L* = 0) to white (L* = 100). The a* value indicates redness ranging from green (a* < 0) to red (a* > 0), and the b* value ranges from blue (b* < 0) to yellow (b* > 0).

Chroma (C*) was calculated according to the equation [41]:

(3) $$C*=\sqrt{{a*}^{2}+{b*}^{2}},$$

The Hue angle (h*) was calculated according to the equation [41]:

(4) $$h*=\tan^{-1}\;\left(\frac{b*}{a*}\right),\;\mathrm{when}\;a*>0,\;b*>0,$$

(5) $$h*=180+\tan^{-1}\;\left(\frac{b*}{a*}\right),\;\mathrm{when}\;a*<0,\;b*>0.$$

#### 2.3.2. Texture Analysis

Garlic texture was assessed using a texture analyzer TA.XT plus (Stable Micro Systems, Godalming, UK). A cylinder P/6 probe was used to determine the stickiness of garlic. The following settings were applied: pre-test speed was 3 mm s^−1^, probe movement speed was 2 mm s^−1^, and post-test speed was 10 mm s^−1^. The sample penetration depth was 2 mm. Ten cloves from different garlic bulbs were analyzed. The analysis results were recalculated to N.

Analyses of the physical properties were performed in 10 replicates.

### 2.4. Statistical Analysis

Data analysis was carried out using Statistica version 7 software (TIBCO Software, Palo Alto, CA, USA). The results were analyzed by using a factorial analysis of variance (ANOVA). The arithmetical means and standard deviations of the experimental data were calculated. Fisher’s Least Significant Difference test (LSD) was applied to the experimental results to assess significant differences between mean values at the significance level of *p* < 0.05.

## 3. Results and Discussions

### 3.1. Changes of BG Chemical Composition

#### 3.1.1. Proximate Composition Changes

The DM content of fresh garlic was 40.76% (Table 2). During the fermentation process, garlic’s DM content increases. The DM content in BG samples ranged from 48.69 to 84.86%, i.e., from 1.2 to 2.1-fold more than fresh garlic. The highest content of DM was determined in BG3 samples, which were fermented at the highest humidity (80%) and the highest temperature (80 °C). BG1 samples were fermented in vacuum bags, and for this reason, moisture could not evaporate, and the amount of DM in BG1 samples differed very slightly from that of WG.

The DM content in BG can differ depending on the heat treatment conditions. Choi et al. [8] found that within 35 days of fermentation at 70 °C and 90% humidity, 35.79% DM in WG can change to 70.12% DM in BG. During processing in the mentioned conditions for 14 days, the amount of dry matter in BG was 68.23%. In our case, the DM content in BG2 samples fermented for 14 days but at 80 °C and 80% humidity, the content was 1.1-fold higher. Kang [10] indicated that during fermentation for 14 days and changing the conditions from 90 °C and 100% humidity to 65 °C and 50% humidity, 37.69% DM in WG changed to 41.52–60.97% DM in BG. Sasmaz et al. [12] found that during garlic fermentation under a controlled temperature of 60–90 °C and humidity of 80–90% between 20 and 60 days, the amount of DM changed from 34.60–39.65% in WG to 32.10–70.49% in BG. Zhang et al. [18] established that DM content after heating BG for 15 days at 80 °C and 80% humidity was 52.88%. Our BG2 samples’ DM content reached almost 1.5-fold. The content of DM in BG depends on the content of DM in WG, but the study’s authors do not report this data. The evaporation of moisture during fermentation and the increase in DM content influenced higher amounts of chemical compounds found in BG samples (Table 2). On the other hand, the fermentation process created the conditions for synthesis and an increase of biologically active substances in BG [8,9,10,12,18,21].

Garlic contains vitamins, especially vitamins C and B [27]. The value of AAC in our WG samples was 8.03 mg 100 g^−1^ (Table 2). Scientists indicate very different AAC in WG, which can vary several times from 4.44 to 21.59 or even to 31.50 mg 100 g^−1^ [42,43]. These different results may be because the AAC depends on many factors, such as genetic properties, meteorological conditions, soil characteristics, and post-harvest conditions [44,45].

Ascorbic acid is one of the most unstable vitamins and is easily destroyed during processing. The greatest losses occur during the processing of raw materials at higher temperatures. However, in the garlic fermentation process, AAC increased from 1.5 to 5.8-fold, depending on the fermentation conditions (Table 2). In BG2 and BG3 samples that were fermented in mesh bags accumulated a higher AAC compared to BG1, which were fermented in vacuum-sealed plastic bags. The higher AAC was established in BG3 fermented at 80 °C and 80% humidity but a short time (14 days). 

Jang and Park [46] found that the AAC increased from 30.29 in WG to 49.67 mg kg^−1^ in BG and from 6.43 to 326.55 mg kg^−1^ in the elephant BG after 15 days of fermentation, changing the temperature from 50 °C to 90 °C. Kim et al. [27] established that total water-soluble vitamin content increased from 6632.91 mg kg^−1^ in WG to 7618.24–9010.44 mg kg^−1^ in BG. The total water-soluble vitamin content of the BG samples fermented at 70 °C and 60% humidity for 60 h was significantly higher.

Since the predominant acid in BG is lactic acid, differences between the samples may have been influenced by the metabolism of lactic acid bacteria. Many studies show that lactic acid bacteria in fermented foods can synthesize a variety of vitamins, such as folic acid, riboflavin, vitamin C, etc. [20]. The stability of vitamin C is also positively affected by the environment’s acidity. Some authors presume that the least reduction of vitamin C might be related to the specific antioxidants accumulated in the product [47]. In the BG samples, some phenolic compounds probably could behave as such protection of vitamin C stability.

Wang et al. [20] stated that vitamins synthesized during the fermentation of lactic acid bacteria can be considered as nutritional fortification of food.

Pacholczyk-Sienicka et al. [48] indicated that fresh garlic accumulates various organic acids, such as malic acid, succinic acid, γ-aminobutyric acid (GABA), lactic acid, fumaric acid, and formic acid. These compounds contribute to the sour and tangy taste of garlic. Titratable acidity indicates the total amount of acidic compounds in the product. Our research has shown that the titratable acidity of WG was 0.12% (Table 2). During the fermentation process, the titratable acidity of BG increased from 1.5 to 2.1-fold compared to WG. Zang et al. [18] found that the total acidity of BG increased with increasing temperature. Our results confirm this statement since the highest acidity was found for BG3 fermented at the highest temperature (Table 2). However, the longer treatment time (28 and 99 days) decreased titratable acidity in BG samples, regardless of the temperature and relative humidity used.

Sasmaz et al. [12] detected the titration acidity of the BG samples ranging from 10.36 to 15.49 g 100 g^−1^ while in the WG samples were 0.75–0.78 g 100 g^−1^. Similarly, Zhang et al. [18] found that WG titratable acidity was 4.6 g kg^−1^ while BG titratable acidity ranged between 33.6 and 37.5 g kg^−1^ depending on the applied heat treatment temperatures from 60 to 90 °C. The formation of short-chain carboxylic acids from hexoses and the degradation of Amadori products and dicarbonyl compounds formed from carbohydrates during thermal processing in the course of caramelization and Maillard reactions result in higher acidity in black garlic [18].

Researchers have reported that new organic acids are formed through complex chemical reactions during the fermentation process [12]. According to Lu et al. [49], lactic acid is formed by the fermentation of BG in hot and humid conditions and is a major organic acid in BG. Therefore, higher titratable acidity might be related to the increase of lactic acid in BG.

The lactic sourness influences the unique taste of BG, and lactic acid, as a strong antioxidant, could have contributed to the antioxidant capacity of BG [49,50].

Garlic has been reported to be a source of bioactive proteins, peptides with various pharmacological and therapeutic potentials, and functional food ingredients [51]. Different cultivars of garlic contain protein from 6.3 to 9.5% [52,53,54]. Our research showed that WG CP content was 3.8% of DM (dry mass) (Table 2). 

CP content in BG might vary between 6.18 and 14.50% [11,55,56]. Sasmaz et al. [12] indicated protein content variations in BG from 6.31 to 14.52% depending on different fermentation conditions. Botas et al. [55] found 7.4% proteins in BG, but the fermentation conditions were not described. Tahir et al. [52] found a CP content of 8.1–9.5% in BG fermented at 65–85 °C and 70 to 80% humidity from 20 to 30 days. Using a long treatment (30, 40, 60, and 90 days) of fermentation at 70 °C, the protein content in BG increased from 6.18 to 7.52% [56].

During fermentation, the amount of CP increased in all tested BG samples from 1.4 to 3.2-fold (Table 2). The highest CP (12.10%) was in the BG3 samples fermented at 80% humidity and 80 °C for 14 days. Dewi and Mustika [56] found an increase in protein content after 60 days of fermentation treatment. The increase in CP content is due to the loss of moisture and increased protein synthesis during fermentation. During a longer fermentation, the CP content in BG begins to decrease, which is confirmed by our data (Table 2). During more than 60 days of fermentation, the amount of CP decreases due to the breakdown of proteins into amino acids and short-chain peptides [12,56].

According to researchers, fresh garlic contains approximately 1.5–2.23% fiber [52,57]. After the analysis, it was found that the CF content in WG was 0.81% of DM (Table 2). 

The different fermentation conditions significantly influenced the amount of CF in the tested BG. Fermentation in mesh bags (BG2 and BG3) at higher than 60 °Cs and 80–85% humidity increased the CF content in garlic the most significantly (almost sevenfold). A lesser increase (about twofold) in CF content was established in BG1 samples fermented in plastic bags at 50 °C and 70% humidity. Tahir et al. [52] had similar results, finding higher CF content in BG from 2.49 to 2.53%, although the increase compared with WG was less (only 0.9–1.1-fold). In our case, treatment time does not significantly affect CF content in BG. 

Scientists also found a significant increase in the total amount of carbohydrates in BG during heat treatment. An increasing amount of carbohydrates indicates a rising amount of polysaccharides, e.g., dietary fibers. Botas et al. [55] found higher values from 1.3 to 1.9-fold of carbohydrate content in the BG samples compared with WG. Tahir et al. [52] established 2.0 to 2.2-fold higher carbohydrate content in BG. Dewi and Mustika’s [56] research showed that the carbohydrate content increased from 18.19 to 45.48% during fermentation from 30 to 90 days at 70 °C.

CA content in different cultivars of garlic from different regions varies considerably from 0.60 to 7.09% of DM [43,52]. The CA content was 0.67% of DM in the WG samples we studied (Table 2). 

The amount of CA in BG is determined to be higher. The remarkable increase in the CA content in BG may be due to the reduced moisture content during fermentation. Tahir et al. [52] found CA content in BG of 0.83–1.70%, with Botas et al. [55] finding 3.2%. Kang [10] determined the CA content in BG to be from 75.36 to 114.98 mg 100 g^−1^—1.5-fold more than in WG. Dewi and Mustika [56] found that CA content varied from 1.65 to 2.72% and stated that ash content increases proportionally due to the increasing period of the fermentation process. The CA content of our studied BG varied between 0.81 and 2.60% of DM, and the highest was found in BG3, where the amount of DM was the highest (Table 2).

The health-promoting effects of garlic are related not only to the high content of vitamins, sulfur-containing compounds, and minerals but also to the phenolic compounds [58]. The amount of phenolics in the fruit and vegetables is greatly influenced by the genotype, growing technologies, and post-harvest storage conditions. However, the most important influence on the amount and composition of phenolic compounds in BG is the fermentation conditions [12,22]. Najman et al. [58] asserted that fermentation processes significantly increase the content of total polyphenols, phenolic acids, and flavonoids. Compared to WG in BG, phenolic compounds increase 3 to 4-fold, or even 5-fold [21,59]. The data provided by different sources on the total content of phenolic compounds in BG varies from 12.35 to 15.12 g kg^−1^ [18], from 66,270 to 81,270 mg GAE 100 g^−1^ [12], 12.50–15.10 mg GAE g^−1^ DM [58], etc.

Our research showed that the TPC in WG was 59.73 mg GAE 100 g^−1^ (Table 2). TPC in BG increased 6.6–9.5-fold compared to WG. According to Choi et al. [8], from the results of total polyphenols and total flavonoids, the optimum aging period of BG to maximize antioxidant content may be the 21st day of aging. In our research, the highest TPC content was significantly in BG3 samples fermented in mesh bags at 80% humidity and 80 °C for 14 days. The BG3 samples established the highest TPC and AAC. In BG2 samples, TPC decreased compared with the BG3. The 99-day-long fermentation period can have a negative impact on the TPC. The lowest increase in the TPC was found in BG1 samples, which were fermented in plastic bags at 70% humidity and 50 °C for 28 days.

#### 3.1.2. The Antioxidant Activity Changes

Phenolic compounds in food products act as antioxidants by reacting with a variety of free radicals. Choi et al. [8] stated that the increase in the antioxidant activities of BG may be due to the increase in total polyphenols, flavonoids, and ascorbic acid contents during the aging period. Sato et al. [26] and Jeong et al. [29] obtained results that confirm that as the TPC increases during garlic fermentation, AA also increases, and BG demonstrates significantly higher antioxidant properties than fresh garlic. The AA of garlic was established according to 2,2-Diphenyl-1-picrylhydrazylradical scavenging activity (DPPH). The AA of the WG analyzed was 6.01% (Table 2). The obtained results are in line with the results of other authors who showed the AA according to DPPH in fresh garlic at the level of 4.65 to 11.22% [8,60,61]. The AA of BG increased from 5 to almost 10-fold depending on fermentation conditions and time, from 32.03 to 59.22% (Table 2). The highest AA increase was established in BG3 samples fermented in mesh bags at 80% humidity and 80 °C for 14 days. In BG2 samples fermented for 99 days, a weaker AA was determined (Table 2). Our data coincides with those of others who analyzed the influence of fermentation time on BG antioxidant activity. Choi et al. [8] stated that BG antioxidant activity, which varied between 37.32 and 74.48%, was highest on the 21st day of fermentation when the content of phenolics increased significantly.

### 3.2. Changes of BG Physical Properties

#### 3.2.1. BG Color Changes

During the garlic fermentation process, which can be more accurately called the thermal process, the sensory properties of the product completely change, i.e., taste, aroma, color, and texture. When garlic is processed at higher temperatures, the cloves’ color from white turns brown and black due to the Millard reaction, known as the non-enzymatic browning reaction [8]. Researchers stated that the enzymatic and non-enzymatic browning reactions are responsible for the change in garlic’s color and the appearance of a sweet and slightly sour taste of black garlic [58,62].

The color parameters of WG depend on the characteristics of the cultivar, growing conditions, etc. According to researchers [9,11], color parameters may vary slightly: L* = 75.99–83.63, a* = −1.71–−2.56, b* = 13.42–18.31, C* = 13.53–8.48, and h* = 97.24–97.95. WG color analysis showed that white, green, and yellow colors prevailed in fresh garlic, accordingly: L* = 66.70, a* = −0.16, and b* = 27.76 (Table 3). In our case, a more intense white color is indicated by a higher C* = 27.76 and a slighter amount of yellowness, h* = 90.33.

Yuan et al. [62] found that the white color of garlic changes already after the first day of fermentation. The longer the garlic is processed, the darker the color becomes. 

Bedrníček et al. [63] found that the L* values of BG fermented for 15 days (82–72 °C) varied from 10.64 to 26.51 according to the garlic cultivar. Meanwhile, a* values ranged from 1.26 to 9.50 and b* values from 1.55 to 10.82. Sasmaz et al. [12] BG color analysis results were different. BG L* values varied from 29.00 to 30.82, a* values from 0.46 to 1.27, b* values from 0.04 to 0.54, C* values from 0.59 to 1.44, and h* values from 13.98 to 33.77. The results were probably influenced by different fermentation conditions, 60–90 °C, 80–90% humidity, and treatment duration from 20 to 60 days.

In our research case, BG samples’ L* value varied from 5.61 for BG3 (fermented at 80 °C) to 12.93 for BG1 (fermented at 50 °C) (Table 3). BG samples differed from WG by the lower values of L*, which means dark color, and the lower b* values, which means lighter yellowness. A positive a* coordinate value in BG samples indicates their redness. BG3 samples were distinguished by the lowest values of a* and b* coordinates, accordingly 1.76 and 3.14, to compare with BG1 and BG2. C* and h* values for BG samples were noticeably lower than the WG samples, indicating that the BG has a lower saturation than WG. Choi et al. [8] pointed out that the BG color changes due to the heat treatment were caused by compounds formed in the initial, intermediate, and final stages of the Maillard reaction.

#### 3.2.2. BG Texture Changes

The typical crunchy texture of fresh garlic completely changes during heat treatment in BG to soft and creamy. Bedrniček et al. [63] stated that the average hardness of fresh garlic samples was 34.29 N, but the BG samples showed 3.01 N hardness, which means an approximately 11-fold decrease. In our case, the hardness of WG was 27.29 N, and the hardness of BG samples varied between 1.26 and 3.12 N (Table 4). Comparing the BG sample’s texture with WG, their hardness decreased 9 to 22-fold. Although the hardness of the analyzed BG samples differed slightly, these differences were insignificant (Table 4).

The food product’s stickiness can be perceived in the palate, teeth, and tongue when it is masticated. The texture of fresh garlic is crunchy when chewed, but the texture of BG becomes soft and sticky. The stickiness we found was −0.03 N for WG and from −0.08 to −0.25 N varied for BG (Table 4). The highest level of stickiness was determined for BG3 samples fermented at 80% humidity and 80 °C for 14 days. BG3 stickiness was probably most influenced by the highest temperature used during the process. Food texture studies show that higher stickiness indicates a collapse of the product structure.

This treatment-induced decrease in hardness and increase in stickiness may be related to changes in cell wall polysaccharides at higher temperatures [63].

## 4. Conclusions

After processing fresh garlic at different temperatures, relative humidity, and different periods, the obtained black garlic differed in its physical properties and chemical composition.

It was established that regardless of the conditions, the chemical composition of black garlic became richer during the fermentation process. Significantly increased vitamin C content (1.5–5.8-fold), titratable acidity (14.7–21.0-fold), protein (1.4–3.2-fold), fiber (4.6–7.0-fold), and ash (1.2–3.9-fold) content, amount of total phenolic compounds (6.6–9.6-fold) and antioxidant activity (5.3–9.9-fold). Fermented garlic turned dark in color and soft and sticky in texture.

The higher fermentation temperature (80 °C) but the shorter time (14 days) had the greatest positive effect on the quality of black garlic. Higher ascorbic acid and fiber content, the highest titratable acidity, protein, ash, total phenolic content and highest antioxidant activity were found in BG3 samples fermented in mesh bags at 80% humidity and 80 °C for 14 days. Fermenting for a longer time, i.e., for 28 or 99 days, decreased the amount of chemical compounds compared to BG fermentation for 14 days. The chemical composition of BG fermented in mesh bags was better than the samples fermented in plastic bags.

## Figures and Tables

**Table 1:** Black garlic fermentation conditions.

Samples	Humidity, %	Temperature, °C	Fermentation Time in Days
BG1	70	50	28
BG2	85	60	99
BG3	80	80	14

**Table 2:** Garlic chemical composition.

Analysis	WG *	BG1	BG2	BG3
DM (%)	40.76 ± 0.20 ^d^	48.69 ± 0.43 ^c^	77.61 ± 0.29 ^b^	84.86 ± 0.39 ^a^
AAC (mg 100 g^−1^)	8.03 ± 1.27 ^c^	11.72 ± 1.27 ^b^	44.70 ± 1.27 ^a^	45.98 ± 0.25 ^a^
TA (%)	0.12 ± 0.03 ^d^	2.06 ± 0.15 ^b^	2.06 ± 0.15 ^b^	2.53 ± 0.10 ^a^
CP (% DM)	3.80 ± 0.03 ^d^	8.53 ± 0.05 ^b^	5.15 ± 0.10 ^c^	12.10 ± 0.01 ^a^
DF (% DM)	0.81 ± 0.01 ^c^	3.75 ± 0.21 ^b^	5.63 ± 0.85 ^a^	5.66 ± 0.43 ^a^
CA (% DM)	0.67 ± 0.05 ^d^	0.81 ± 0.05 ^c^	2.40 ± 0.03 ^b^	2.60 ± 0.04 ^a^
TPC (mg GAE 100 g^−1^)	59.73 ± 1.10 ^c^	394.19 ± 6.64 ^b^	402.00 ± 22.52 ^b^	571.75 ± 66.67 ^a^
AA (%)	6.01 ± 1.33 ^d^	46.78 ± 1.37 ^b^	32.03 ± 1.16 ^c^	59.22 ± 0.37 ^a^

* WG—white garlic (control variant), BG1, BG2, BG3—black garlic. Significant differences (*p* < 0.05) in lines are marked by different letters; for each measured parameter, the general mean ± SD is presented.

**Table 3:** Garlic color analysis results.

Analysis	WG **	BG1	BG2	BG3
L*	66.70 ± 0.27 ^a^	12.93 ± 0.41 ^b^	10.24 ± 0.15 ^c^	5.61 ± 0.19 ^d^
a*	−0.16 ± 0.21 ^d^	11.96 ± 0.38 ^a^	2.85 ± 0.01 ^b^	1.76 ± 0.20 ^c^
b*	27.76 ± 0.47 ^a^	13.83 ± 0.57 ^b^	4.97 ± 0.05 ^c^	3.14 ± 0.20 ^d^
C*	27.76 ± 0.49 ^a^	25.77 ± 0.45 ^b^	5.73 ± 0.03 ^c^	3.6 ± 0.20 ^d^
h*	90.33 ± 0.67 ^a^	49.24 ± 0.54 ^c^	60.11 ± 0.61 ^b^	60.67 ± 0.61 ^b^

** WG—white garlic (control variant), BG1, BG2, BG3—black garlic. Significant differences (*p* < 0.05) in lines are marked by different letters; for each measured parameter, the general mean ± SD is presented.

**Table 4:** Garlic texture analysis results.

Analysis	WG *	BG1	BG2	BG3
Hardness (N)	27.29 ± 3.39 ^a^	1.26 ± 0.06 ^b^	3.12 ± 0.13 ^b^	1.85 ± 0.19 ^b^
Stickiness (N)	−0.03 ± 0.01 ^a^	−0.08 ± 0.01 ^b^	−0.09 ± 0.01 ^b^	−0.25 ± 0.01 ^c^

* WG—white garlic (control variant), BG1, BG2, BG3—black garlic. Significant differences (*p* < 0.05) in lines are marked by different letters; for each measured parameter, the general mean ± SD is presented.

## Data Availability

The original contributions presented in the study are included in the article, further inquiries can be directed to the corresponding author.
